# Exposure to Seasonal Temperatures during the Last Month of Gestation and the Risk of Preterm Birth in Stockholm

**DOI:** 10.3390/ijerph120403962

**Published:** 2015-04-10

**Authors:** Ana M. Vicedo-Cabrera, David Olsson, Bertil Forsberg

**Affiliations:** Department of Public Health and Clinical Medicine, Occupational and Environmental Medicine, Umeå University, SE901 87 Umeå, Sweden; E-Mails: david.olsson@envmed.umu.se (D.O.); bertil.forsberg@envmed.umu.se (B.F.)

**Keywords:** preterm birth, climate change, ambient temperature, heat, cold

## Abstract

Recent evidence from studies performed mainly in warm climates suggests an association between exposure to extreme temperatures late in pregnancy and an increased risk of preterm delivery. However, there have been fewer studies on the effect of low temperatures. The aim of this study is to explore the potential association between both heat and cold during late pregnancy and an increased risk of preterm birth in the northern location of Stockholm, Sweden. All singleton spontaneous births that took place in greater Stockholm (1998–2006) were included. Non-linear and delayed effects of mean temperature on the risk of preterm birth were explored through distributed lag non-linear models. Extreme and moderate heat and cold were estimated separately through quasi-Poisson regression analysis in two seasonal periods (heat in warm season, cold in cold season). The risk of preterm birth increased by 4%–5% when the mean temperature reached the 75th percentile (moderate heat) four weeks earlier (reference: the annual median value), with a maximum cumulative risk ratio of 2.50 (95% confidence interval: 1.02–6.15). Inconsistent associations were obtained for cold and extreme heat. Exposure to moderately high temperatures during late pregnancy might be associated with an increase in risk of preterm birth in Stockholm.

## 1. Introduction

Effects of climate change on population health have barely been investigated but are a major public health concern. One of the emerging research issues gaining attention is the effect of exposure to high or low temperatures during pregnancy. A few recent studies addressed the potential association between the exposure to extreme temperatures and different birth outcomes, including preterm birth, low birth weight, stillbirth, and congenital anomalies [1,2,3,4,5,6,7,8,9]. It is known that pregnant women might be more susceptible to temperature extremes due to the extra physical and mental strain of pregnancy and their limited ability to maintain temperature balance [10,11]. Several studies with animal models observed direct effects on embryo and fetus development associated with maternal hyperthermia [12]. In fact, the fetus has a limited ability to regulate temperature, and it is entirely dependent upon the mother’s thermoregulatory capacity [12]. However, to date the population of pregnant women has not been yet considered as a vulnerable group in the current Heat Health Warning Systems in force.

Preterm birth, referring to all births with gestational age < 37 complete weeks [13], is the leading cause of perinatal mortality and morbidity worldwide [14,15], and gestational age at delivery is one of the key determinants of fetal maturity at birth [16]. It is reported that preterm infants are more likely to experience adverse health outcomes during childhood and later in life [16,17,18,19]. In recent decades, great efforts have been made to reduce the burden of disease due to prematurity, mainly by identifying potential risk factors in different populations. Along with several fetomaternal characteristics, recent studies have suggested an association between preterm delivery and environmental factors such as meteorological variables and air pollution levels [20,21,22]. Short-term effects of both high and low temperatures have been observed in several studies performed in different settings [2,3,6,7,9,23], but in other studies this association seemed unclear [24,25]. To our knowledge, only one study explored the effects of a very cold climate using data from the early twentieth century [23].

## 2. Methods

### 2.1. Data Collection

Data on all deliveries by women residing in the greater Stockholm area from 1998 to 2006 were extracted from the Swedish Medical Birth Register. The number of inhabitants in the greater Stockholm area is about 1.6 million per 3500 km^2^, with approximately 40% of the population residing in the city proper.

We restricted the analysis to all singleton births whose labor had not been artificially induced and whose reported gestational age was between 22 and 42 weeks. Gestational age was estimated through ultrasound examinations in more than 98% of the births in the Swedish Medical Birth Register. For the remainder of the cases, gestational age was calculated according to the mother’s last recalled menstrual period.

Greater Stockholm is located in northern Europe and borders the Baltic Sea. It has a temperate climate with very mild summer temperatures and cold winters. During the study period, daily air temperature and humidity data were derived from the monitor located in Central Stockholm and managed by the City of Stockholm Environment and Health Administration.

Daily 24-h mean concentrations of particulate matter (PM) with aerodynamic diameter < 10 µm (PM_10_), nitrogen dioxide (NO_2_), and daily maximum eight-hour running mean of ozone (O_3_) were calculated using the hourly measurements taken from an urban background (rooftop) station located in Central Stockholm managed by the City of Stockholm Environment and Health Administration.

### 2.2. Statistical Analysis

We performed a time-series analysis where the main outcome was the daily count of preterm births. Poisson generalized additive models accounting for overdispersion were combined with distributed lag non-linear models (DLNM) [26], and these were applied to assess the non-linear and delayed contribution of temperature on preterm birth up to four weeks before the delivery.

A modified pregnancies-at-risk approach was applied to properly account for the short-term variations of the population at risk [9]. This consisted of introducing an offset with the daily count of all pregnancies at risk of being preterm on a specific day (that is, all gestations that were between the 22nd and 36th week on a specific day). A correction term was also included that represents the daily distribution of the gestational age of the pregnancies included in the offset, with the aim of weighting the counts by the probability of giving birth conditional on the gestational age. The rationale for doing this is that higher gestational age increases the likelihood of giving birth, thus the potentially different distribution across days would give a variable baseline probability of preterm births that could affect our short-term association estimates [27].

**Figure A1:**
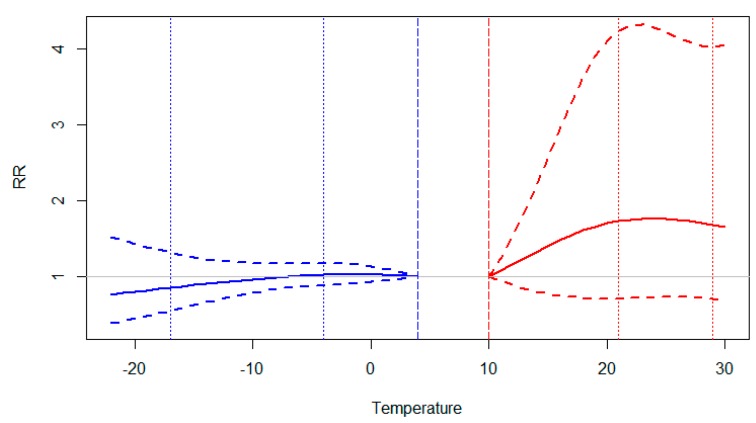
Cumulative risk ratio (RR, 95% confidence interval) of preterm birth up to 30 days for maximum daily temperature (red) and minimum daily temperature (blue) estimated for the warm and cold seasons, respectively. Vertical dashed lines, from right to left: 1st and 25th percentile of minimum daily temperature during the cold season (dotted, blue) (−18 °C; −4 °C), median minimum daily temperature for the annual series (dashed, blue) (4 °C), median maximum daily temperature for the annual series (dashed, red) (10 °C), 75st and 99th percentile of maximum daily temperature during the warm season (dotted, red) (21 °C; 29 °C).

Notes: The model applied was the same as the one used in the main analysis for mean temperature, and according to q-AIC, the selected specifications for the cross-basis dimensions (natural spline-natural spline, with 2df in each).

Daily mean temperature was used as the main exposure metric to explore the contribution of heat and cold on the risk of preterm birth. This decision was made with the aim of simplifying the approach and was based on the similarity to the results obtained in the preliminary analysis for mean, maximum, and minimum temperature (Figure A1). To do this, our study period was split into two different seasons according to their similarity in monthly mean-temperature distributions; the warm season was defined as May to September, and the cold season as October to April. In both cases we obtained eight seasonal sub periods, from May 1998 (upper limit) to September 2005 (lowest limit) for warm season, and from October 1998 to April 2006 for cold season. We did not include the 2006 warm season because the offset of pregnancies-at-risk began to decrease gradually from approximately mid-August 2006 (22 weeks before 31 December 2006, that is, the last date of inclusion of pregnancies). In other words, new pregnancies-at-risk were not incorporated in the offset upon this date because of the truncation of the series at the end of 2006. Thus, we obtained risk estimates associated with high temperatures when the analysis was restricted to the warm season and the corresponding risk estimates of low temperatures when the analysis was restricted to the cold season.

In each case, we obtained two different association estimates according to the range of temperature explored: the moderate heat (and cold) that was estimated in terms of risk ratio (RR) and its 95% confidence interval (CI) for the increase (or decrease) in daily mean temperature from the annual median value to the 75th percentile of the warm season distribution (or 25th percentile of the cold season). Additionally, RR estimates were obtained for the extreme heat and cold expressed as the change in risk when mean daily temperatures reached the 99th/1st percentile (using as reference value the 75th/25th percentile, respectively) of the corresponding season. In the main analysis, we reported the results as lag-specific RR estimates and the overall cumulative RR up to 30 days (calculated as the sum of the contributions of each lag-specific RR up to this day).

According to DLNM methodology, mean daily temperature was introduced in all models as a cross-basis term. Two natural cubic spline functions with two degrees of freedom (df) were specified to accurately capture the relationship between temperature and the outcome, and across the lag-specific estimates up to 30 days, similarly to previous studies [9]. The combination of the number of df in each function was selected according to quasi-Akaike Information Criterion (q-AIC), choosing between 2, 3, 4, and 5 df, where the corresponding internal knots were placed at equally spaced percentile values of temperature (by default) and at equally spaced intervals in the log scale of lags.

We controlled for long-term trends and seasonality through two penalized cubic splines with 1 df per year and month in each season (testing between 0.8, 1, 2, 3 df per month or year). This was a conservative method, but it had enough flexibility to properly control for confounding. Also, factor variables such as the day of the week and holidays were introduced in the model along with a non-linear term of mean relative humidity (as a penalized cubic spline with 5 df of the moving average of two consecutive days). Again, the number of df was selected according to the q-AIC.

Several additional analyses were performed to better characterize the estimated associations obtained in the main analysis. We obtained the week-specific cumulative RR for moderate/extreme heat and cold using the initial definitions of warm and cold seasons and restricting each season period to the hottest and/or coldest months. That is, we defined a restricted warm season from June to August and the corresponding cold season between November and March.

We also compared the cumulative RR estimates of the main model with the ones obtained when air pollutants were considered. We introduced each air pollutant (O_3_, NO_2_, PM_10_) one by one in the main model for temperature effect as a linear term of the moving average of the previous week. Additionally, we tested the linearity of this air pollution term comparing the fitting of the initial approach (using a linear term) with the fitting when a non-linear term was introduced (penalized cubic spline with 10 df) using the ANOVA Fisher test. Finally, we explored how the air pollution coefficient varied when mean temperature (as a cross-basis term) was or was not included in the model.

Analyses were performed with STATA version 11 software (StataCorp LP, College Station, Texas) and the DLNM package in the R software version 2.15.1 (R Development Core Team, 2012) [26].

## 3. Results

During the entire study period (from 1998 to 2006), a total of 134,802 births took place in the greater Stockholm area. However, only 95,069 births (70.5%) met the inclusion criteria for the present study, with an overall proportion of preterm birth during the study period of 3.5% (Table 1). A higher number of births were included in the cold season analysis, but the estimated mean numbers of births per month in each season were similar with 915 and 910 births per warm and cold months, respectively. Likewise, we obtained a slightly higher incidence of preterm births during the cold season (3.7% *versus* 3.3% in the warm season).

**Table 1:** Description of the distribution of total and preterm births included in the study per period.

Period	Total Births	Preterm Births N (%)
Annual	95,069	3,321 (3.5%)
Warm season	36,577	1,204 (3.3%)
Restricted warm season	21,065	706 (3.2%)
Cold season	51,052	1,878 (3.7%)
Restricted cold season	35,555	1,349 (3.8%)

Notes: Warm season (May–September, 1998–2005). Restricted warm season (June–August, 1998–2005). Cold season (October–April, 1998–2006). Restricted cold season (November–March, 1998–2006).

As shown in Table 2, the median value of the daily mean temperature in the entire annual series was 7.7 °C, whereas the corresponding values for the warm and cold season periods were 15.2 °C and 2.0 °C, respectively. Extreme temperatures in greater Stockholm ranged from 25.6 °C, the maximum value of daily (24 h) mean temperature registered during the study period, to the minimum value of −21.5 °C. Similar humidity patterns were observed between the different seasonal periods, with a median value around 77% in the annual series, and 83.0% in the cold season and 70.2% in the warm season. Several differences in correlation between the daily levels of air pollutants and mean temperature and humidity were observed in each season (Table 3). We obtained a moderate correlation coefficient between mean temperature and O_3_ (r = 0.3318) and PM_10_ (r = 0.2495) in the warm season, but almost no correlation for NO_2_ (r = −0.0685). In the cold months, NO_2_ seemed to be negatively correlated with temperature (r = −0.3044). Higher correlation coefficients were obtained between air pollutants and relative humidity, for example, for O_3_ in both seasonal periods and for PM_10_ in the cold season.

**Figure 1:**
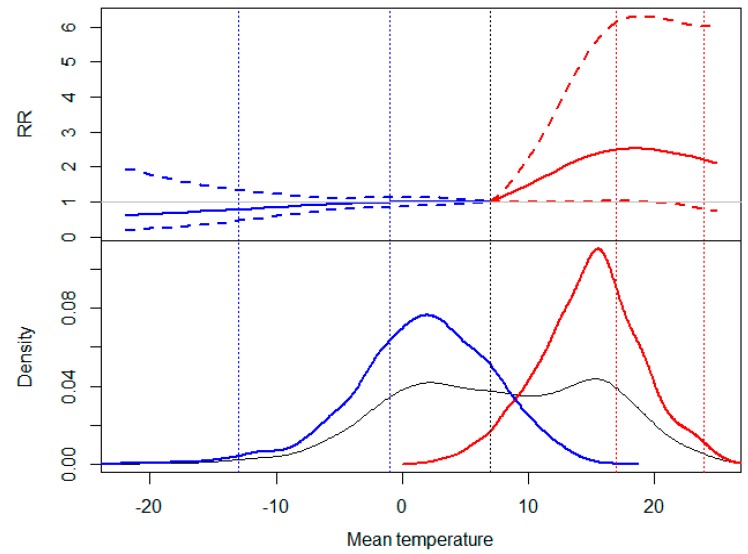
Top: Cumulative risk ratio (RR) and 95% confidence interval of preterm birth up to 30 days by mean temperature. Bottom: kernel distribution graphs of the mean temperature (Grey: entire annual period; Red: warm season; Blue: cold season). Vertical dashed lines in different mean temperature values: 1st and 25th percentile of the cold season (–13.1 °C; –1.5 °C), mean value of the entire annual series (black, long dash) (7.7 °C), and 75th and 99th percentile of the warm season (17.5 °C; 24 °C).

According to Figure 1, we observed different association patterns between high and low temperatures and the risk of preterm birth (as cumulative RR up to 30 days). A positive and statistically significant slope of heat was obtained for moderately high temperatures registered during the warm season, with a maximum cumulative RR of 2.50 [95% CI: 1.02–6.15] when mean temperature reached the 75th percentile. Consistent results were not observed for the range of extremely high temperatures. On the other hand, a slightly negative but inconsistent association was obtained for cold temperatures, with no noticeable changes in the slope between the two temperature intervals.

**Figure 2:**
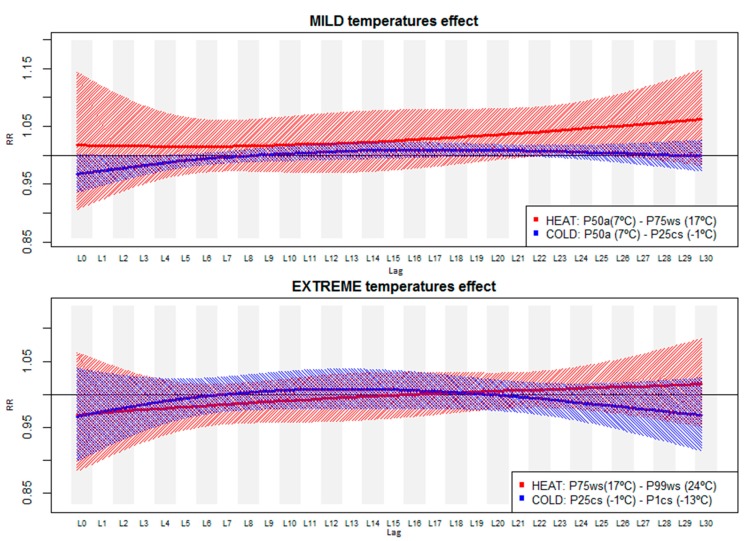
Lag-specific risk ratios (RR) and 95% confidence interval of the effect of moderate mild (top) and extreme (bottom) heat and cold up to 30 days after the exposure (a: defined percentile of the annual mean temperature distribution; CS/WS: defined percentile of mean temperature distribution in the cold or warm season).

Our results suggest different distribution patterns for the contribution of heat and cold on the risk of preterm birth across the 30-day lagged period (Figure 2). In Table A1, the numerical RR estimates (and 95% CI) of this figure are reported. We obtained a delayed heat effect when temperature increased up to the 75th percentile (moderate heat interval) during the warm season consisting of an increased risk of preterm birth of 4%–5% from the 22nd to the 28th day after the exposure. For moderately low temperatures, despite obtaining negative but non-significant association estimates in the first week after the exposure, only a small increased risk was obtained during the third week of exposure (RR from lag16 to lag24 of 1.01 [95% CI: 1.00–1.02]). Again, consistent results were not obtained for an increase or decrease in extreme temperatures (from the 25th/75th to the 1st/99th percentile) (Figure 2, bottom).

**Table 2:** Descriptive statistics of the environmental variables in Stockholm (1998–2006).

Environmental Variables		Minimum		1th Percentile		25th Percentile		Median		75th Percentile		99th Percentile		Maximum	
**Meteorological conditions**															
*Mean temperature* *(°C)*	Annual	−21.5		−11.6		1.5		7.7		14.5		23.1		25.6	
	WS/WSr	2.5	8.9	5.4	10.2	12.4	14.8	15.2	16.7	17.5	18.88	24.0	24.28	25.6	25.58
	CS/CSr	−21.5	−21.5	−13.1	−14.2	−1.5	−2.8	2.0	0.2	5.6	3.0	12.7	8.5	15.5	10.5
*Relative humidity (%)*	Annual	34.6		43.7		67.0		77.8		85.8		94.6		97.5	
	WS/WSr	34.6	34.6	39.6	39.6	61.0	61.3	70.2	69.8	78.1	78.0	92.5	92.3	96.0	93.9
	CS/CSr	42.8	51.5	48.2	55.3	74.3	77.8	83.0	84.4	87.7	88.7	94.4	94.4	96.9	96.3
**Air pollution levels**															
*NO_2_* *(µg/m^3^)*	Annual	2.4		5.0		12.0		16.7		21.5		39.6		70.8	
	WS/WSr	3.2	3.2	4.3	4.7	10.3	9.6	14.0	13.4	18.4	17.5	34.2	31.2	43.2	43.2
	CS/CSr	3.1	4.4	5.7	6.2	14.2	14.8	19.0	19.7	23.7	24.3	41.9	42.1	70.8	70.8
*O_3_* *(µg/m^3^)*	Annual	7.2		21.6		51.6		64.4		78.2		114.8		143.4	
	WS/WSr	23.8	25.6	34.4	35.2	60.3	61.6	72.1	71.7	84.2	84.1	117.1	117.9	126.5	125.8
	CS/CSr	7.2	7.2	19.5	19.0	46.2	44.5	57.6	55.9	70.6	67.5	110.7	92.8	127.1	107.4
*PM_10_* *(µg/m^3^)*	Annual	2.2		4.1		9.3		12.8		18.4		48.6		87.6	
	WS/WSr	3.3	3.5	4.8	4.1	9.3	9.0	12.1	11.5	15.7	14.7	42.2	33.7	67.0	55.7
	CS/CSr	2.2	2.2	3.8	3.8	9.1	8.8	13.2	12.6	20.6	18.9	49.1	46.6	87.6	76.3

Notes: WS: warm season (May–September, 1998–2005). WSr: restricted warm season (June–August, 1998–2005). CS: cold season (October–April, 1998–2006). CSr: restricted cold season (November–March, 1998–2006).

**Table 3:** Pearson correlation coefficients estimated between the corresponding daily environmental variables per season (grey shade for warm season; black for cold season).

	Mean Temperature	Mean Relative Humidity	NO_2_	O_3_	PM_10_
Mean temperature	1	−0.1637	−0.3044	0.1346	0.183
Mean relative humidity	−0.1559	1	0.1552	−0.681	−0.438
NO_2_	−0.0685	0.1097	1	−0.2929	0.157
O_3_	0.3318	−0.4571	−0.1277	1	0.404
PM_10_	0.2495	−0.1205	0.2905	0.4748	1

**Figure 3:**
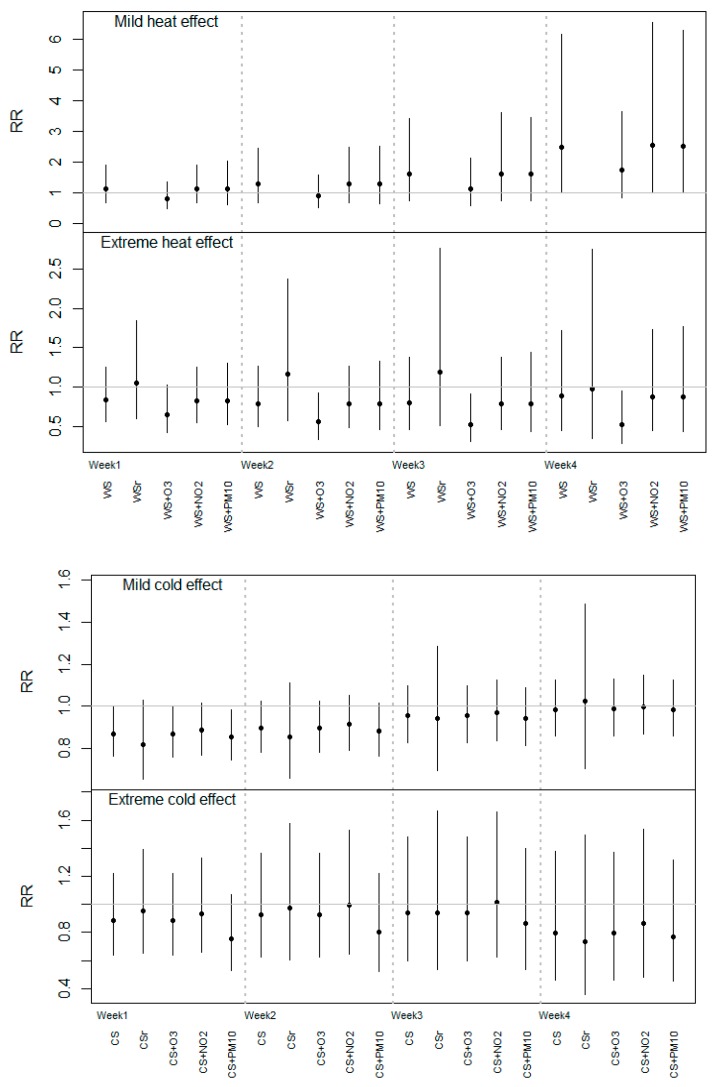
Cumulative risk ratios (RR) estimated up to the end of each week (from the 1st to 4th week after the exposure) for moderate/extreme heat and cold effects in the different models.

Notes: WS/CS: estimates per season with the main temperature model; WSr/CSr: estimates using the same main model applied in the restricted warm (June–August) and cold (November–March) seasons, respectively. WS/CS + air pollutants (linear terms of moving average level of the previous week).

In Figure 3, the heat and cold estimates are shown in terms of the cumulative RR estimated at the end of each week (from week 1 to week 4) with the different models explained in the Methods Section. If we only considered the results of the main analysis (labeled as “WS”) for heat, we did not observe any consistent cumulative RR of extreme temperatures in the different weeks, whereas for the moderate interval an increasing trend in RR estimates was obtained from week 1 to week 4. The only statistically significant results were for the last week, which corresponds to the reported result in Figure 1. Similar estimates were obtained for moderate heat, except when O_3_ (“WS + O_3_”) was included in the model, which produced RR values that were slightly smaller compared to the main model estimates. The same pattern was observed for the extreme heat estimates, but in this case the risk values in the O_3_ model were below 1 and statistically significant, and it remained constant throughout the four weeks. As expected, when we restricted the analysis to the hottest months, higher but not statistically significant RR estimates were obtained for extreme heat. The RR of moderate heat was not estimated for this restricted period due to the lack of statistical power (insufficient number of days with moderate mean temperature).

Focusing on the results from main analysis for cold (Figure 3, bottom, labeled as “CS”), we observed a positive trend of the cumulative RR for moderate temperatures from week 1 to week 4, but in this case, statistically significant risk values below 1 were obtained for the 1st week after the exposure (RR = 0.87 [95% CI: 0.76–0.99]), and any RRs for extreme cold effect again reached significance. On the other hand, similar association estimates were obtained if we restricted our analysis to the coldest months or when O_3_ and NO_2_ were considered in the model. However, the cumulative RR changed slightly towards zero for extreme cold when PM_10_ was included (“CS + PM_10_”) in weeks 1, 2, and 3 compared to the “CS” estimate.

Finally, the results shown in Table A2 suggest that there was no direct association between air pollutants *per se* and the risk of preterm birth, even if we did not consider temperature in the model.

## 4. Discussion

This study explored the association between the risk of preterm birth and the exposure to heat and cold during late pregnancy in a northern population in Europe. Our results suggest that exposure to moderate heat during the previous four weeks before the delivery might be associated with an increased risk of giving birth prematurely. However, no changes in risk were obtained for extreme heat or cold temperatures. The present study provides more evidence about this public health issue, which is gaining the attention of the main entities involved in health protection (for example, the World Health Organization, Intergovernmental Panel on Climate Change) in the face of climate change. However, the still-unknown mechanisms triggering preterm delivery, along with the heterogeneity among study populations and climate conditions, make the assessment of the role of ambient temperature as a risk factor for preterm birth challenging.

We observed different heat and cold association patterns, with non-significant changes in the risk of preterm birth due to low temperatures, which is in agreement with the results of Schifano *et al.* [6]. A recent study also performed in a Swedish population suggests similar exposure-response relationship between high and low temperatures and risk of preterm deliveries [23]. However, they included birth data from the beginning of the twentieth century and, in contrast to our approach, they used as the exposure variable the average temperature during the entire gestation period.

With regard to high temperatures, several investigations in both European and non-European populations reported an immediate increase in the risk of preterm birth during the following days after the exposure to high temperatures or heat waves, with consistent effects lasting up to one week [6,7,9,28,29]. In contrast, our results suggest a more delayed increased risk of up to four weeks after the exposure to moderate heat, but not for short-term exposures, nor for extreme temperatures. We did not observe any noticeable difference in the risk estimates for maximum and/or minimum temperature with the results for mean daily temperature (Figure A1). This supports the idea that extreme temperatures (or episodes), which might be better captured by maximum and minimum daily temperatures, were not found to be associated with increased risk of preterm birth. We note that most of the previous study settings were predominantly in warm climates, and so the reported immediate effects referred to temperatures that were clearly above the range corresponding to the moderate heat (from 7°C to 17 °C), and even the extreme interval (from 17°C to 24 °C), in Stockholm. Another reason that might explain this effect for moderate heat, and not for extreme temperatures, might be the fact that people usually stay outdoors longer when the ambient temperature is moderate because this temperature range might be considered as less harmful than extreme temperatures.

In light of our results, one might suggest that this delayed estimate of moderate temperatures would be related to a different underlying mechanism that might trigger preterm delivery. We propose several potential explanations for this delayed effect. First, it has been reported that one of the multiple risk factors of preterm delivery is the development of an inflammatory process due to an intrauterine infection, accounting for near 40% of all cases of premature birth [30]. We argue that this span of one month between exposure and preterm birth might coincide with the time needed for the infection to develop, that is, from the colonization of the microorganisms to the initiation of the inflammatory response [31]. Thus, it might be possible that the exposure to warm temperatures might trigger this chain of events that concludes with the initiation of a preterm delivery. Another possible reason could be the association of elevated temperatures and changes in blood pressure in specific exposure windows during pregnancy. Because most of the preterm cases were in the 35th or 36th week of gestation (70% of the preterm births in our population), the exposure to moderate temperatures might have occurred when these preterm cases were in their 31st or 32nd gestational week (four weeks earlier). We argue that during this gestational window pregnant women might be more susceptible to even small increases in temperatures, and in fact, it is reported that from the beginning of the third trimester (from the 28th week onwards) maternal blood pressure begins to increase [32].

In the present study, the exposure to cold was not found to be associated with a higher risk of preterm birth, which is contrary to the results reported in a Swedish study where extreme cold adversely affected preterm birth [23]. As stated before, the comparability of those results with the ones obtained in this work is limited because they used 100-year-old data and the temperature indicator corresponded to the mean exposure levels during the entire gestation period; however, we cannot disregard the possibility that cold temperatures might have an effect if the exposure had occurred prior to late pregnancy. It would be interesting to identify in future work the critical exposure window *in utero*, beyond the short-term effect, when the exposure to defined ranges of temperature might alter the health status of the pregnant women and subsequently trigger preterm delivery.

We performed several additional analyses in order to shed more light on our results. We restricted the heat and cold analyses to the hottest and coldest months and, as expected, higher RR estimates were obtained in the extreme heat from the first week after exposure (Figure 3). However, as previously stated, due to the reduction in the length of the series and the resulting loss of statistical power, these effects did not reach statistical significance. This fact would suggest that the effect of occasionally very hot days, but not the whole season, might be more immediate than the moderate heat effect. This fact is contrary to the results from a similar study conducted in Valencia, Spain, where moderate heat was associated with an immediate increase in risk of preterm birth, while a more delayed effect (two weeks after exposure) was obtained for extreme temperatures during the warm season [9]. In other studies, the effect of a heat wave or extreme temperatures on preterm birth were obtained during the first week after the exposure [6,28]. These contrasting results show the difficulty in generalizing location-specific temperature effects to other regions due to potential acclimatization of the population to different climates.

We observed a slight decrease in RR estimates when weekly average O_3_ levels were included in the heat-effect model and when PM_10_ was considered in the cold-effect model compared to the main results. Because we did not observe any effect of both air pollutants on the risk of preterm birth by themselves (Table A2), it might be possible that in the case of O_3_ this change in RR estimate might be due to the fact that both variables are moderately correlated during the warm season (Table 3). Also, if we control for O_3_ we are subtracting the effect that temperature exerts on the risk of preterm birth through the generation of O_3_. In fact, a recent commentary discussed the potential inappropriateness of controlling for air pollutant levels in temperature models because this might be an intermediate variable between temperature and the outcome [33]. Regarding PM_10_, it seems that change in RR was only observed in the very low temperature interval and during the first and second week after the exposure. The correlation between both variables was very low, even considering only the lowest range of temperatures (r = 0.0430); therefore, the joint effect between temperature and PM_10_ should be further addressed in future works.

To our knowledge, this is the first study performed in a cold climate that explores the short-term effects of heat and cold on preterm birth. As stated previously, the consequences of exposure to both high and low temperatures during gestation have been addressed in a limited number of studies compared to the number of studies exploring the association between birth outcomes and other environmental variables such as air pollution. Likewise, a wide heterogeneity in the results has been observed across the different study locations, perhaps due to the general characteristics and habits of the population and acclimatization. This limits the generalizability of the conclusions derived in the different studies. In addition, there is no agreement in several methodological aspects, such as the explored exposure window, the definition of the study population, heat or cold indicators, *etc*. Thus, new research performed in defined or unexplored locations contributes useful evidence. In order to continue advancing knowledge on the relationship between temperature and preterm birth, it would be necessary to perform new studies on this issue in other locations and using a common methodological framework.

In the present study, we performed a time-series analysis that enabled us to control for the underlying seasonal trend in conceptions that, if not controlled for, might have introduced bias in our temperature-effect estimates. We also took into account the potential short-term variations of the population at risk by applying the modified version of the pregnancies-at-risk approach. This consists of including in the model not only the daily count of ongoing pregnancies-at-risk at each day (between the 23rd and 36th gestational week), but also the daily distribution of the gestational age of this set-at-risk as a way to control to the variable baseline risk of giving birth depending on the week of gestation. Likewise, the influence of time-invariant confounding variables, such as individual maternal characteristics or habits, is controlled by design. Also, our estimates would not be affected by other unmeasured confounders that might vary across time (*i.e*., maternal smoking behavior), mainly because these would need to fluctuate in the same time scale as temperature does (day by day) in order to confound our results.

We restricted the analysis to all spontaneous births as a way to exclude planned deliveries and to better evaluate the effect of temperature as a trigger for preterm birth, as was done in other studies [2,7]. However, the establishment of these inclusion criteria means that we considerably reduced the sample size of our study population. The number of preterm birth cases per day with these characteristics was relatively low (the mean daily count was 1.05 (SD: 1.05) preterm births) compared to other recent studies [2,3,6]. This fact limits our ability to obtain robust effect estimates, as can be observed from the borderline results and wide confidence intervals.

## 5. Conclusions

In conclusion, one of the key elements for improving protection of the population from temperature effects, and more generally to climate change, is to identify specific vulnerable subgroups for whom targeted prevention measures should be addressed. We observed an increased risk of giving birth prematurely after exposure to moderate heat during the last month of gestation, which suggests that pregnant women might be considered a subpopulation that is potentially susceptible to heat. Despite our small and in some cases inconsistent estimates, it should be taken into account that even small effects of a ubiquitous exposure on an important health outcome can have large public health impacts. Given the extensive contribution of preterm delivery to infant mortality estimates worldwide and the considerable impact on premature newborns’ health later in life, our results, along with the evidence obtained from previous studies, should be considered for the design of future policies combatting climate change.

## Appendix

**Table A1:** Lag-specific risk ratio and 95% confidence intervals of the effect of heat and cold in moderate and extreme temperatures.

Lag	Moderate Heat Effect	Extreme Heat Effect	Moderate Cold Effect	Extreme Cold Effect
1	1.02 [0.91–1.14]	0.97 [0.88–1.06]	0.97 [0.94–1.00]	0.97 [0.9–1.04]
2	1.02 [0.92–1.12]	0.97 [0.90–1.05]	0.97 [0.95–1.00]	0.97 [0.91–1.03]
3	1.02 [0.93–1.10]	0.97 [0.91–1.04]	0.98 [0.96–1.00]	0.98 [0.93–1.03]
4	1.02 [0.95–1.09]	0.98 [0.93–1.03]	0.98 [0.96–1.00]	0.98 [0.94–1.03]
5	1.02 [0.96–1.08]	0.98 [0.94–1.02]	0.99 [0.97–1.00]	0.99 [0.95–1.02]
6	1.02 [0.97–1.07]	0.98 [0.95–1.02]	0.99 [0.98–1.00]	0.99 [0.96–1.02]
7	1.02 [0.97–1.06]	0.98 [0.95–1.02]	0.99 [0.98–1.00]	1.00 [0.97–1.02]
8	1.02 [0.97–1.06]	0.98 [0.95–1.02]	1.00 [0.99–1.01]	1.00 [0.97–1.03]
9	1.02 [0.97–1.06]	0.99 [0.96–1.02]	1.00 [0.99–1.01]	1.00 [0.98–1.03]
10	1.02 [0.97–1.06]	0.99 [0.96–1.02]	1.00 [0.99–1.01]	1.00 [0.98–1.03]
11	1.02 [0.97–1.07]	0.99 [0.96–1.02]	1.00 [0.99–1.02]	1.01 [0.98–1.03]
12	1.02 [0.97–1.07]	0.99 [0.96–1.03]	1.00 [0.99–1.02]	1.01 [0.98–1.04]
13	1.02 [0.97–1.07]	0.99 [0.96–1.03]	1.01 [0.99–1.02]	1.01 [0.98–1.04]
14	1.02 [0.97–1.07]	1.00 [0.96–1.03]	1.01 [0.99–1.02]	1.01 [0.98–1.04]
15	1.02 [0.97–1.08]	1.00 [0.96–1.03]	1.01 [0.99–1.02]	1.01 [0.98–1.04]
16	1.02 [0.97–1.08]	1.00 [0.96–1.03]	1.01 [1.00–1.02]	1.01 [0.98–1.04]
17	1.03 [0.98–1.08]	1.00 [0.97–1.03]	1.01 [1.00–1.02]	1.01 [0.98–1.03]
18	1.03 [0.98–1.08]	1.00 [0.97–1.03]	1.01 [1.00–1.02]	1.00 [0.98–1.03]
19	1.03 [0.98–1.08]	1.00 [0.97–1.03]	1.01 [1.00–1.02]	1.00 [0.98–1.03]
20	1.03 [0.99–1.08]	1.00 [0.97–1.03]	1.01 [1.00–1.02]	1.00 [0.98–1.03]
21	1.04 [0.99–1.08]	1.00 [0.98–1.03]	1.01 [1.00–1.02]	1.00 [0.98–1.02]
22	1.04 [1.00–1.08]	1.01 [0.98–1.03]	1.01 [1.00–1.02]	1.00 [0.97–1.02]
23	1.04 [1.00–1.08]	1.01 [0.98–1.03]	1.01 [1.00–1.02]	0.99 [0.97–1.02]
24	1.04 [1.00–1.09]	1.01 [0.98–1.04]	1.01 [1.00–1.02]	0.99 [0.97–1.02]
25	1.05 [1.00–1.09]	1.01 [0.98–1.04]	1.01 [0.99–1.02]	0.99 [0.96–1.02]
26	1.05 [1.00–1.10]	1.01 [0.98–1.05]	1.00 [0.99–1.02]	0.99 [0.95–1.02]
27	1.05 [1.00–1.11]	1.01 [0.97–1.05]	1.00 [0.99–1.02]	0.98 [0.95–1.02]
28	1.05 [1.00–1.11]	1.01 [0.97–1.06]	1.00 [0.98–1.02]	0.98 [0.94–1.02]
29	1.06 [0.99–1.13]	1.01 [0.96–1.07]	1.00 [0.98–1.02]	0.98 [0.93–1.02]
30	1.06 [0.99–1.14]	1.01 [0.96–1.08]	1.00 [0.98–1.02]	0.97 [0.92–1.02]

**Table A2:** Effect of air pollution levels (moving average of the last 7 days) on the risk of preterm birth by season, when the cross-basis term of mean temperature is included or not in the model, and the corresponding quasi-Akaike value (q-AIC). Effect reported in percent change per 10 µg/m^3^ increase in air pollutant levels.

Air Pollutant	Warm Season		Cold Season	
	% Change [95% CI]	q-AIC	% Change [95% CI]	q-AIC
O_3_	−1.76 [−8.58–5.12]	3146.7	1.04 [−4.02–6.13]	4671.0
O_3_ + Mean temperature (cross-basis)	7.75 [−2.02–17.61]	2528.9	−0.21 [−5.46–5.07]	4610.9
PM_10_	−7.07 [−20.01–6.05]	3145.7	−0.68 [−8.63–7.34]	4671.1
PM_10_ + Mean temperature (cross-basis)	0.90 [−23.45–25.85]	2530.7	−3.73 [−12.23–4.84]	4610.0
NO_2_	−8.19 [−29.07–13.15]	3146.4	−11.68 [−25.53–2.37]	4667.8
NO_2_ + Mean temperature (cross-basis)	1.63 [−26.28–30.34]	2530.7	−6.87 [−23.42–9.97]	4610.0
